# Human Sirt-1: Molecular Modeling and Structure-Function Relationships of an Unordered Protein

**DOI:** 10.1371/journal.pone.0007350

**Published:** 2009-10-08

**Authors:** Ida Autiero, Susan Costantini, Giovanni Colonna

**Affiliations:** 1 CRISCEB (Interdepartmental Research Center for Computational and Biotechnological Sciences) Second University of Naples, Naples, Italy; 2 CROM (Oncology Research Centre of Mercogliano) “Fiorentino Lo Vuolo”, Mercogliano, Italy; 3 Department of Biochemistry and Biophysics, Second University of Naples, Naples, Italy; Cardiff University, United Kingdom

## Abstract

**Background:**

Sirt-1 is a NAD+-dependent nuclear deacetylase of 747 residues that in mammals is involved in various important metabolic pathways, such as glucose metabolism and insulin secretion, and often works on many different metabolic substrates as a multifunctional protein. Sirt-1 down-regulates p53 activity, rising lifespan, and cell survival; it also deacetylases peroxisome proliferator-activated receptor-gamma (PPAR-γ) and its coactivator 1 alpha (PGC-1α), promoting lipid mobilization, positively regulating insulin secretion, and increasing mitochondrial dimension and number. Therefore, it has been implicated in diseases such as diabetes and the metabolic syndrome and, also, in the mechanisms of longevity induced by calorie restriction. Its whole structure is not yet experimentally determined and the structural features of its allosteric site are unknown, and no information is known about the structural changes determined by the binding of its allosteric effectors.

**Methodology:**

In this study, we modelled the whole three-dimensional structure of Sirt-1 and that of its endogenous activator, the nuclear protein AROS. Moreover, we modelled the Sirt-1/AROS complex in order to study the structural basis of its activation and regulation.

**Conclusions:**

Amazingly, the structural data show that Sirt-1 is an unordered protein with a globular core and two large unordered structural regions at both termini, which play an important role in the protein-protein interaction. Moreover, we have found on Sirt-1 a conserved pharmacophore pocket of which we have discussed the implication.

## Introduction

The sirtuin family is widely distributed from archaea and eubacteria to eukaryotes and seven different homologous proteins are found in the humans that show a central highly conserved region, defined as the catalytic core [1]. In comparison to other proteins of the family, Sirt-1 presents two large regions, i.e. the amino and carboxyl terminals, that are missing in all the other sirtuins. Sirt-1 is a NAD+-dependent deacetylase closely related to yeast *Sir2*, the first gene discovered in sirtuin family, which has NAD+ dependent class III histone deacetylase activity [2]. Sites of phosphorylation and SUMOylation consensus were recently found also in the amino- and in carboxyl-terminal regions, and these were proposed having regulation and localization functions [3].

Experimental data support the Sirt-1 implication in processes including chromatin remodelling, transcriptional silencing, chromosomal stability, cell cycle progression, apoptosis, autophagy, metabolism, growth suppression, inflammation, and stress response [4]. In fact, the Sirt-1 regulation activity occurs through the deacetylation reaction of various and different substrates such as p53 [5], forkhead box class O (FOXO) transcription factors [6], peroxisome proliferator activated receptor (PPAR)g co-activator 1a (PGC-1α) [7], nuclear factor (NF)-kB and others, which are closely linked to some age-related diseases [5]. Also Sirt-1 stimulates eNOS activity and increases the endothelial NO. Its inhibition in the endothelium of arteries inhibits endothelium dependent vasodilation and decreases bioavailable NO [8].

Moreover, it was seen that Sirt-1 is a negative modulator of adipogenesis by docking with the nuclear receptor co-repressor (NcoR) [9] and induces a decrease of pro-inflammatory cytokine release [10] and a promotion of carcinogenesis [11] by the negative control of Nuclear factor-kB (NF-kB). Experimental data demonstrated that the inhibition of NF-kB in hepatocytes in vivo promotes hepatocarcinogenesis.

Kaeberlein *et al.* demonstrated firstly the anti-aging effects of *Sir2* showing that in *Saccharomyces cerevisiae* the integration of extra copies of *Sir2* extended lifespan up to 30% [12]. Similar effects of *Sir2* were subsequently observed in *C. elegans* and *Drosophila melanogaster* [13]–[14]. In fact, the overexpression of *Sir-2* [15] increased lifespan up to 50% in *C. elegans* but in *Drosophila*, an extra copy of the *Sir2* gene extended lifespan in female and male by 29 and 18%, respectively.

Because Sirt-1 deacetylates non histone proteins, including various transcription factors, it is involved in the control of important biological mechanisms. Through its catalytic activity, it exhibits diversified functions in cell type-specific manner, which have pathophysiological implications in cancer, obesity, inflammation and neurodegenerative diseases [4], [16]–[17]. Its modulation leads an increase in mitochondrial biogenesis, and an improvement of glucose metabolism in mitochondria but also in skeletal muscle and adipose tissues [18]. These evidences suggest that Sirt-1 could be a novel target to treat metabolic disorders such as type 2 diabetes.

Numerous experimental data [2], [19]–[20] have shown the modulation of the catalytic activity of Sirt-1 exerted through its allosteric effectors. Kim et al (2007) demonstrated that the nuclear protein AROS is an endogenous activator of Sirt-1 which increases its activity interacting with the allosteric site. Accordingly with the functional relevance of Sirt-1 activation, recent works focused on its interaction with small allosteric effectors [19]–[21].

In particular, the phenolic compound, named resveratrol, is the first found allosteric activator, that has been reported to extend lifespan in *yeast* [21], *Caenorhabditis elegans*, *Drosophila* [22] and rodents [20]. It improves the metabolism and glucose tolerance, as well as the overall physical performance in various stress tests [7]. However recent efforts have identified new compounds significantly more potent than resveratrol [19]–[20].

Even if the substrates and the effects of Sirt-1 activation have been well characterized, no structural information about its activation and regulation is known.

On the basis of the biological importance of this protein, we focused our attention to create a model of the whole structure of human Sirt-1 in order to understand its interaction with the allosteric effectors and to propose the molecular basis of its activation and regulation.

## Results

### Modelling of the catalytic core and allosteric site of Sirt-1

**Figure 1:**
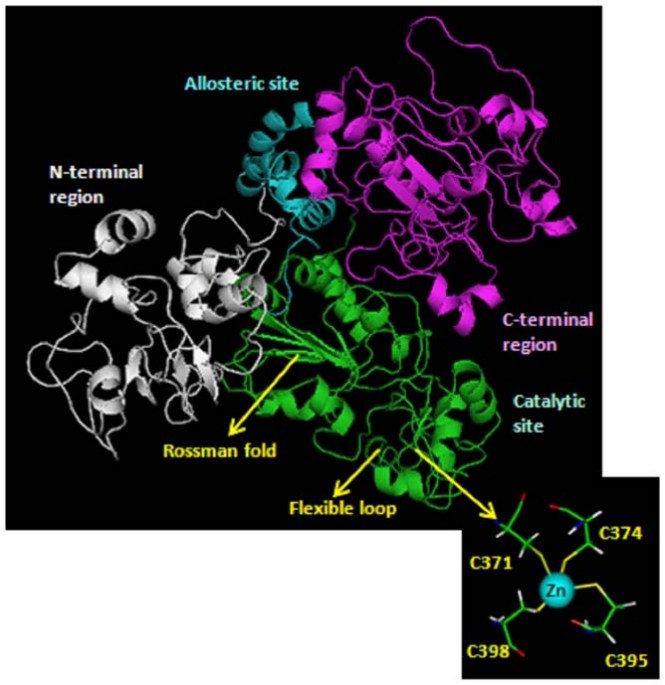
Model of human Sirt-1. The different structural/functional regions are shown in different colors: in white the N-terminal region, in cyan the allosteric site, in green the catalytic core and in magenta the C-terminal region. The region of the zinc binding (down on the right) shows the four tetrahedrically coordinated cysteines.

The best model has been obtained for the catalytic core of human Sirt-1 using as template the structures of human Sirt-2 [23], Hst2 from Saccharomyces cerevisae [21] and Sir2-Af1 from Archaeoglobus flugidus [22] is shown in Figure 1. This model has 94,9% residues in the most favoured regions and a Prosa Z-score of 8,7. These values, compared also with those of the template structures, indicate that a good quality model has been created.

**Figure 2:**
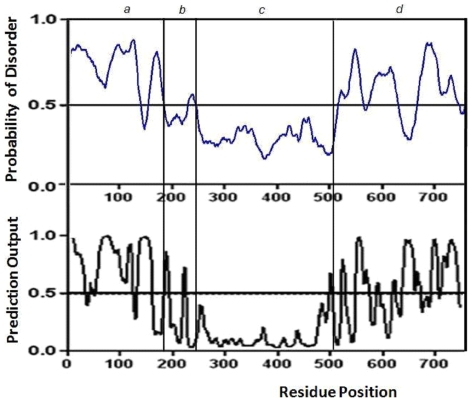
Predictions of disorder regions. Top - The disorder predictions (blue) made by DISOPRED for the N-terminal region (a), the allosteric site (b), the catalytic core (c) and the C-terminal region (d). Bottom - The prediction of disordered binding regions (black) made by Anchor.

**Figure 3:**
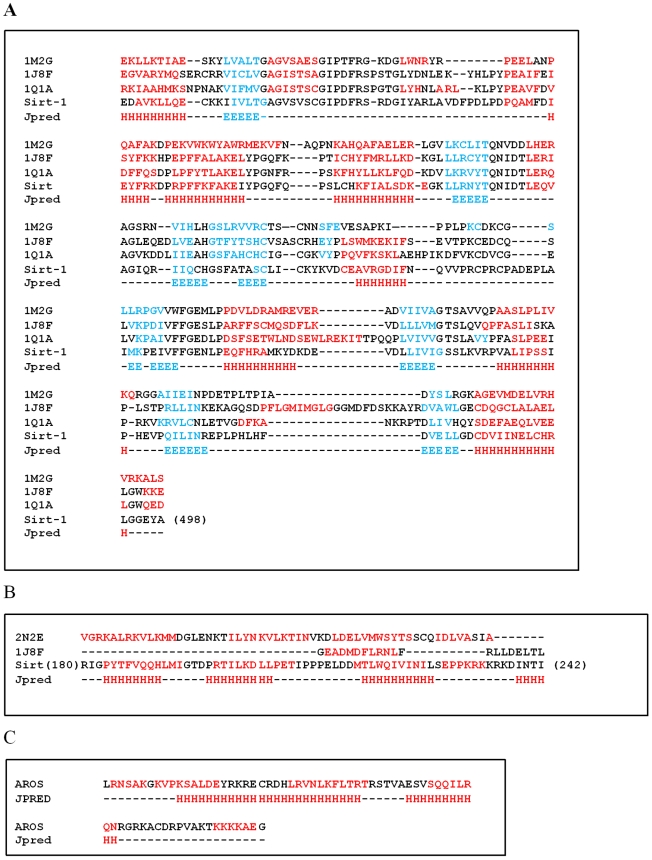
Primary and secondary structures of Sirt-1 and AROS. a) catalitic core (from residue 244 to 498), b) allosteric site (from residue 181 to 243) and c) AROS. Helix and beta strands regions are shown in red and cyan, respectively. Moreover, JPred predictions are reported.

The catalytic core appears well structured in agreement with its low propensity to the disorder, as shown in Figure 2. Secondary structure predictions made by JPRED program agree with the structure of catalytic core obtained by comparative modeling (Figure 3). The core (from residue 244 to 498) is well structured. The model (Figure 1) shows the two structural domains typical of the sirtuin family of which the first one is a Rossman fold with the characteristic β-α-β motives of NAD proteins, and the second one is a smaller sub-domain where the zinc is located. At the interface between domains there is the binding site for NAD. The NAD molecule fits in a specific pocket constituted of a hydrophobic patch on the small sub-domain, and a hydrophilic patch on the large domain. In particular, the NAD molecule presents the adenine and ribose moieties inside the pocket, as described for other sirtuins [22]–[25], but the nicotinamide group is near to the residues 269–295 of human Sirt-1. These residues (indicated with a white arrow in Figure 1) represent another conserved region that forms a flexible loop near the pocket. This loop was proposed as a “frontwall” [25] of the C-site, or a “ceiling”[25] on the pocket. Its flexible organization allows a structural rearrangement of the catalytic domain during the NAD binding, which seems essential for the catalysis. This central role is demonstrated by the orientation of this region in the crystallized sirtuins. In fact, the crystallized complexes with the NAD+ molecule show a same order orientation, but without NAD, this loop region shows a more poorly defined or disordered orientation [21], [23]. This suggests that the NAD+ binding influences the orientation of this flexible loop by triggering the assembly and disassembly of the C pocket [25] as well as the loop's orientation is a measure of the accessibility into the pocket for the nicotinamide or other small inhibitors [1].

The zinc ion is tetrahedrally coordinated by the thiol groups of four Cys residues (at positions 371, 374, 395, 398) which are folded into a single structural unit. This tetrad of cysteine residues are that is broadly conserved in the small sub-domain of all the sirtuin family [23]. and this small zinc-binding domain (see Figure 1) is thought to play a role in substrate-specific binding by the sirtuin proteins [26]. In the classic zinc finger, one zinc atom is bound to two cysteines and two histidines while in this case we have a Cys4-Zn. This structure is composed by about 30 residues included in a flexible structural environment that sterically favours the tetrahedrally coordination of the cysteines.

The allosteric site (from residue 181 to 243) is straddling between the N-terminal and the compact globular core of the protein and shows an all-alpha structure composed by four alpha helices in agreement with secondary structure and disorder predictions (Figure 2b and 3).

Both the catalytic core and the allosteric site have compact and globular shapes according to the definition of globularity recently published [27] with a score of 5.1 and 4.3, respectively.

**Figure 4:**
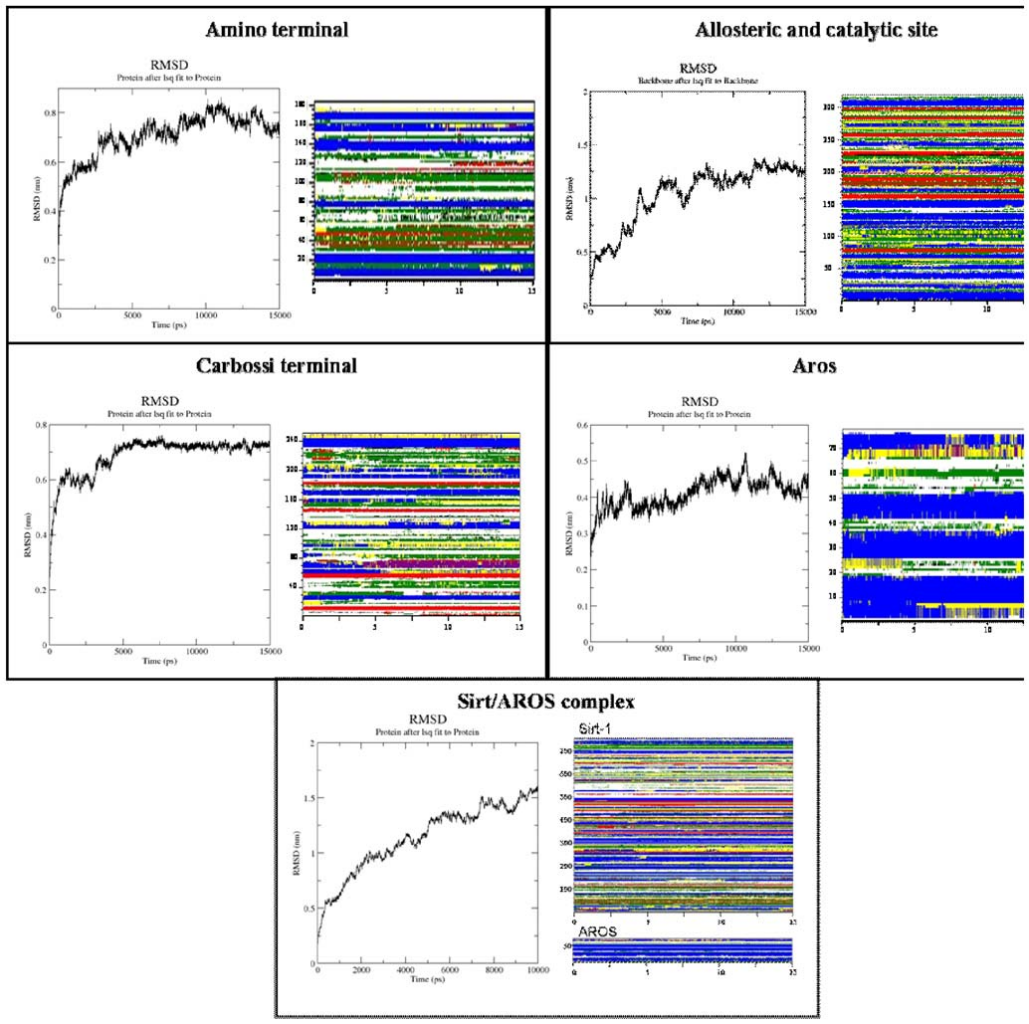
Molecular dynamics results. RMSD evolution and analysis of the secondary structures during the molecular dynamics.

The two models were linked, as reported in Methods, by a flexible loop and subjected to molecular dynamics simulation. The state of equilibrium was reached after 8 ns simulation. The structure remained very stable during the whole simulation time, as confirmed by all the indicators commonly used to analyse MD simulations (Fig. 4A). In particular, the Rossman fold, as well as the smaller domain and the four helices of the allosteric site were well conserved during the simulation. Only two flexible loops, i.e. the final loop of the allosteric site and the “frontwall” in the catalytic domain, presented some fluctuation. An evaluation of the distance between the allosteric site and the catalytic core was obtained in terms of center of mass distances along the trajectories. This distance decreased of 17 Å after 10 ns of simulation and was maintained constant during the last 5 ns of simulation.

### Modeling of the N-terminal and C-terminal regions

Various programs for predicting the secondary structure of globular proteins were unable to have a good consensus for the N-terminal and C-terminal segments of Sirt-1. They have differently predicted in amount and in sequence strings the scattered presence of non alfa and non beta regions in both terminal zones. This observation raised the suspect that the Sirt-1 could be an unordered protein and more appropriate algorithms were used. The N-terminal and C-terminal regions were found largely unordered by two specific structural tests. DISOPRED, a software devoted to the search of unordered regions predicted long segments in N-terminal region (1–150 and 160–182) as well as in C-terminal region (510–580, 585–640 and 680–740). Moreover, the Anchor program, in addition, was also able to predict the presence of definite protein binding regions [28] in the unordered segments (Figure 2 and Table S1). The N-terminal and C-terminal regions were modelled as reported in Methods and resulted made of six very short helices inserted with seven short β-strands and of five short helices inserted with five short β-strands, respectively. The remaining large part of residues are in irregular conformations according to the predictions.

The two modelled regions were independently subjected to molecular dynamics simulations with the same protocol used for the allosteric and catalytic sites. The N-terminus reached a stable equilibrated state after 8 ns and the C-terminal after 4 ns of simulation, respectively. Both models looked very stable (Fig. 4B and C) and the secondary structures present in both models were well kept during the simulation time.

### Complete model of Sirt-1

We created a whole model of the human Sirt-1 by comparing modelling using as templates the previously obtained models of N-terminal, allosteric site, catalytic site and C-terminal site. The model was minimized according to our recent papers [29].

The resulting model has 87.8% residues in the most favoured regions and a Prosa Z-score of -6.57.

The various structural parts that compose the global architecture of the protein are shown in Figure 1. The N-terminal and C-terminal regions are positioned behind the catalytic core, but the groove site of Sirt-1 is exposed to the solvent to receive its substrates. Distances among the centers of mass of catalytic core, N-terminal and C-terminal regions were evaluated. In details, the catalytic core-N-terminal distance resulted to be 38 Å, and that between the catalytic core and C-terminal of 48 Å. The catalytic core exchanges 2 hydrogen bonds and 8 salt bridges with the N-terminal site, and 5 hydrogen bonds and 13 salt bridges with the C-terminal site.

These values confirm that the three regions have distinct structural roles even if one can easily hypothesize that their very close relationships suggests an involvement in the functional activities of the Sirt-1. In fact, the allosteric site is positioned between the N- terminus and the catalytic core in the best position to regulate the enzymatic activity of Sirt-1. Moreover, the N-terminal and C-terminal sites are positioned in a region that does not prevent the catalytic core activity, and this confirms that they have a role of regulation and localization of Sirt-1.

### AROS model

AROS is the endogenous protein that is able to increase the Sirt-1 activity [2], interacting with its allosteric site. Its model was obtained by fold recognition strategy and presents an all-alpha fold composed from 4 alpha helices in agreement with the secondary structure predictions made by JPred [30] (Figure 3C) and with the structural class prediction made by PRECLASSPRO server [31].

The AROS model was subjected to molecular dynamics simulations by using the protocol reported in the Methods. A stable state was reached after 7 ns of simulation time and was kept constant up to the end of the dynamics (Figure 4D).

### Sirt-1/AROS complex

**Figure 5:**
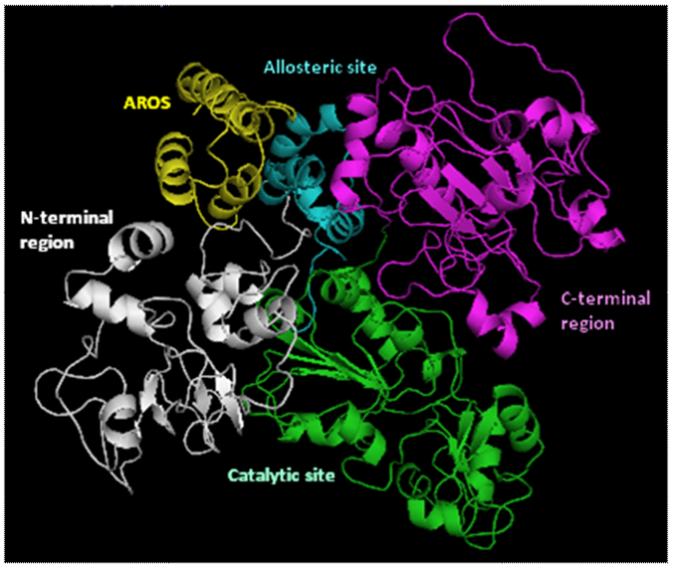
Model of human Sirt-1/AROS complex.

**Figure 6:**
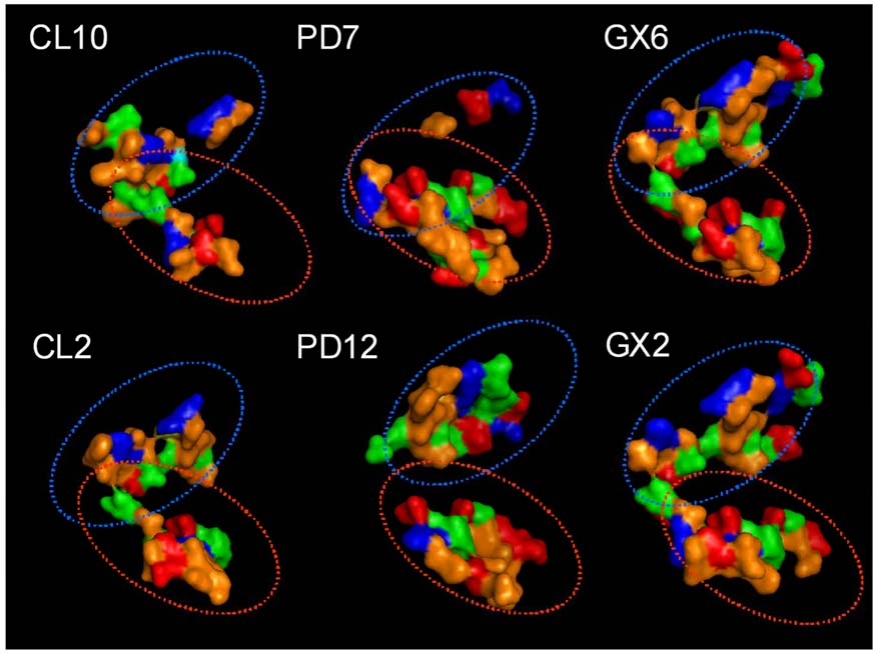
Analysing the Sirt-1/AROS binding groove. The surface of Sirt-1 binding groove of the selected complexes between AROS and Sirt-1, obtained using CLUSPRO (CL10 and CL2), PATCHDOCK (PD7 and PD12) and GRAMMX (GX6 AND GX2) servers. The colours correspond to the chemical properties of each residue. The acid residue are coloured in red, the basic residue are coloured in blue, the hydrophobic residues are coloured in orange and the hydrophilic residues are coloured in green. The oval lines indicate the basic top and the acid bottom, respectively.

AROS is the endogenous protein that is able to increase the Sirt-1 activity [2], interacting with its allosteric site. To investigate the best binding-groove of Sirt-1 for allosteric effectors we used the principal docking servers (i.e. PATCHDOCK, GRAMMX, CLUSPRO) to obtain a set of possible complexes. We firstly selected the complexes having AROS near to the allosteric site because experimental truncation data have suggested that some amino acids of the allosteric site are important for the binding of allosteric activators [19]. It is worthy of note that in the most part of simulated complexes, AROS is placed in the region between the N-terminal site and the allosteric site (Figure 5). For a more detailed analysis, we selected two of the best complexes for each set, in terms of energy binding, interface ASA, number of atoms and residues at the interface, number of hydrogen bonds and salt bridges (see Table 1). Moreover, we compared, in terms of physical-chemical and geometric properties, the exposed residues of Sirt-1 to the binding pocket in each complex to identify pharmacophore features. As shown in Figure 6, the binding groove presents basic residues at the top of the cavity, acidic residues at its bottom and hydrophobic residues as edges.

**Table 1:** The selected complex obtained using CLUSPRO, PATCHDOCK and GRAMMX server.

Complexes	Binding free energy	Interface ASA	Interaction Residues	H-bonds	Salt Bridges
**CL2**	−9.96	1023.49	34	4	24
**CL10**	−10.19	973.9	30	1	29
**PD7**	−10.51	1231.39	34	7	39
**PD12**	−8.92	1230.1	39	4	29
**GX2**	−10.73	1471.8	48	5	11
**GX6**	−12.65	1332.27	40	4	12

For each one are reported the value of: energy binding (Kcal/mol), interface ASA (Å^2^), number of atoms and residues at the interface, number of hydrogen bonds and salt bridge are reported.

The best Sirt-1/AROS complex was obtained using the CLUSPRO web server [32] that uses one of the best docking algorithm tested in CAPRI experiments with a success rate of about 71% [32]. The good quality of this complex is also suggested by the very low value of its binding free energy (−10.19 Kcal/mol), calculated by the DCOMPLEX server [33]. For this complex, we have evaluated the interacting residues, the number of interchain H-bonds and salt bridges and the interface surface area (Tables 2 and 3). The AROS-Sirt-1 complex shows that AROS is located behind to the catalytic groove. This can be considered one of the most favourable structural site to increase the enzyme activity by conformational changes without penalizing the correct interaction between Sirt-1 and its substrates. In particular, AROS and Sirt-1 chains should form one H-bond and 6 salt bridges at their interaction surface. At the interface Sirt-1 also exposed four aromatic residues, one positively charged residue and five negatively charged residues. The interface region of AROS is composed by six and three positively and negatively charged residues, respectively. These data suggest that the predominant interaction between Sirt-1 and AROS is on electrostatic basis and that the four aromatic residues structurally closely in Sirt-1 might play an important role to favour the stacking interactions with other allosteric activators, as well as organic compounds.

**Table 2:** Analysis of the best Sirt-1/AROS complex in terms of interface surface area (Å^2^), interchain H-bonds and number of interaction residues and salt bridges.

	Interface ASA	Interchain H-bonds	Interaction residues	Salt bridges
**Sirt-1**	973.90	1	30	6
**AROS**	906.00	1	25	6

**Table 3:** List of interaction residues between AROS and Sirt-1.

	Interaction residues
**Sirt-1**	MET1; ALA2; **ASP3**; LEU7; **GLU161**; **ASP166**; SER169; HIS170; ALA171; SER172; SER173; SER174; **ASP175**; **TRP176**; PRO184; **TYR185**; **PHE187**; VAL188; HIS191; LEU192; ILE194; GLY195; THR196; **ASP197**; THR219; **TRP221**; GLN222; ILE223; **TRP624**; **ARG627**; VAL628.
**AROS**	**ASP24**; HIS25; LEU26; **ARG27**; **GLU42**; SER43; VAL44; GLN46; GLN47; ILE48; LEU49; **ARG50**; GLN51; **ARG53**; VAL62; **LYS64**; **LYS66**; **LYS69**; ALA70; THR73; VAL74; **GLU77**

The charged residues are evidenced in bold and those aromatic are also underlined.

### Molecular dynamics of Sirt-1/AROS complex

The Sirt-1/AROS complex was subjected to molecular dynamics simulations with the same protocol previously used. The complex reaches a stable equilibrated state after 7 ns of simulation and after that it remains stable. Even the secondary structures were kept enough stable during the simulation.

We have compared the conformation of AROS/Sirt-1 complex before and after the molecular simulation to verify how the mutual interactions changed. We inserted an acetyl-lysine and a NAD molecule in the AROS/Sirt-1 complex using as template the crystallographic structure of *Saccharomyces cerevisae* Sirt-1. Then, we focused our attention on the catalytic groove. We noted that, during the molecular dynamics, a conformational change of the protein occurs mainly in active site region. In details, the residues His 363, Asn 346, Ser 265, Gly 261 and Pro 447, indicated as important for its catalytic role [34], are sterically changed (see Figure S1). Also Pro 447, that makes Van der Waals contacts with the aliphatic portion of the acetyl-lysine side chain and may affect the positioning of residues that contact the acetyl group, is more close to the Acetyllisine while four other residues (His 363, Asn 346, Ser 265, Gly 261) that affect the NAD orientation, approaches to the NAD.

Therefore these results could indicate that the molecular dynamics improves the interaction between Sirt-1 and AROS by some structural parameters (see Table S2) even if longer time scale will be used for studying this type of long range coupling.

## Discussion

Human sirtuin-1, an oxidative stress-response and chromatin-silencing factor, is an unordered protein. About half of its sequence appears to be in a disordered state with long flexible poorly structured regions observed at N- and C-terminus. The modeling has also evidenced a large central globular part very well structured made by two close regions, the catalytic core and the allosteric site. Therefore, the protein can be considered composed by four different regions: N-terminal domain, allosteric site, catalytic core and C-terminal domain. The catalytic core is a central highly conserved structured region common to all the sirtuin family [1] which has the role of catalyzing the NAD^+^-dependent deacetylation reaction, involved in various nuclear events such as transcription, DNA replication, and DNA repair. The core is made by a well organized Rossman fold typical for NAD-dependent proteins. In details, the acetyl lysine substrate has been proposed to bind in a narrow channel that terminates near to the nicotinamide ribose. Moreover, the binding of acetyl-substrate is believed to mediate bending of the nicotinamide ring of NAD+ in a strained conformation also referred to productive NAD+ binding, promoting the cleavage of the ribosyl–nicotinamide bond [21], [24].

In this domain we find an atom of Zinc tetrahedrically coordinated with four cysteine residues also present in the other members of the sirtuin family. The allosteric site is a small structural domain made of four helices and interacting with the core. It is sited in a structural location where it can easily exert a control of the catalytic activity by conformational changes. Several articles report the presence of activators modulating the functional activity of the human sirtuin. One of the activators, the resveratrol, decreases the Michaelis constant of SIRT1 for both the acetylated substrate and NAD+, and increases cell survival by stimulating SIRT1-dependent deacetylation of p53 [35]. Moreover, a small nuclear protein, AROS (**A**ctive **R**egulator **O**f **S**irtuin), has been found to be the first known endogenous active modulator of SIRT1 which directly regulates SIRT1 function. AROS enhances SIRT1-mediated deacetylation of p53 both in vitro and in vivo, and it inhibits p53-mediated transcriptional activity. It is interesting to observe that AROS was unable to inactivate p53 when was used an AROS-binding-defective SIRT1 mutant. This clearly indicates a direct interaction of AROS with a specific site of Sirt-1. Our best model of AROS presents an all-alpha fold composed from 4 alpha helices. We found that AROS binds the same allosteric site of some small synthetic compounds (*not shown; manuscript in preparation*) proposed as putative therapeutics for the treatment of type 2 diabetes [2], [19].

The catalytic core and the allosteric site make a central structured domain (about from residues 181 to 498), compact and globular as assessed by various globularity indices.

Moreover Sirt-1 presents two long disordered structural segments at the terminal regions, missing in the other six homologous proteins belonging to the same family. The N-terminal region is 180 residues long and that C-terminal 249. These regions were predicted largely disordered by DISOPRED program that resulted one of the best algorithm for the accuracy of disorder prediction in CASP7 [36]–[37]. Figure S2 shows the secondary structure of the best model of Sirt-1 calculated by DSSP algorithm that evaluates the Φ and ▒ dihedral angles of each residue and the backbone Hydrogen bonds. From this table as one can see both termini are characterized by numerous and large unstructured regions even if in the C-terminal more structured segments are presents.

Many proteins or protein domains show an intrinsic inability to form a well defined tertiary structure. This property is encoded in their sequence owing to the local depletion of typically buried amino acid residues as well as enrichment of typically exposed amino acid residues (about 40%) (see Table S3). Amino acid residues located in highly mobile regions of protein also possess the smallest volumes and molecular weights as well as the lowest hydrophobicities and the highest flexibility (see Figures S3, S4, S5 and S6). Only in this way the protein can be so dynamically flexible to minimize unfavorable pairwise contacts.

In the last years it has become evident that there is a large number of proteins that do not require a stable structure even under physiological conditions in order to fulfil their biological role [38]–[40]. The importance of protein disorder is underlined by the abundance of partially or fully disordered proteins that are encoded in higher eukaryotic genomes [36] and are involved in many important biological functions [38], which complement the functional repertoire of globular proteins [41]. Disordered segments often act as flexible linkers between folded domains in multidomain proteins [38] and their function is often that of binding specifically other proteins, DNA or RNA through a process, termed coupled folding and binding, that involves a transition disordered-ordered with stable secondary and tertiary structural elements [38]. This “coupled binding and folding” confers several functional advantages in certain types of molecular interactions that often are essential for signaling processes. The high propensity of their residues to stay disordered makes these regions predominantly not structured and this can favour their putative functionality.

In fact, recent papers have shown the importance in Sirt-1 of various phosphorilations sites, nuclear localization signals (NLS) and nuclear export signals (NESs, amino acids ) that were mainly found in the terminal regions [3], [42], [43]. These authors suggested that these regions seem to be involved in regulating enzyme activity and have the peculiarity of being present only in Sirt-1. Moreover, this fact also suggests that C- and N-terminal regions of the human Sirt-1 might be involved in a more fine regulating role in order to exploit its biological mechanisms. In the disordered regions specific protein binding sites were identified by using Anchor program [28]. The prediction of these binding sites is based on estimating the energy content in free and in the bound states, and identifying segments that are potentially sensitive to these changes. In particular, Anchor program has predicted in human Sirt-1 fourtheen disordered binding regions of which four located in the N-terminal domain, two in the allosteric site and other eight in the C-terminal domain (Figure 2-down and Table S1). The two disordered binding regions predicted in the allosteric site agree with those evidenced in Sirt-1/AROS complex (see Table 3). All these data indicate that human Sirt-1 is an unordered protein and that its terminal domains can play different roles. The inherent flexibility of the two termini suggests that the protein has a malleable interface that can allow the binding of several partners or adopt different conformations, as manifested by its high binding capability.

**Figure 7:**
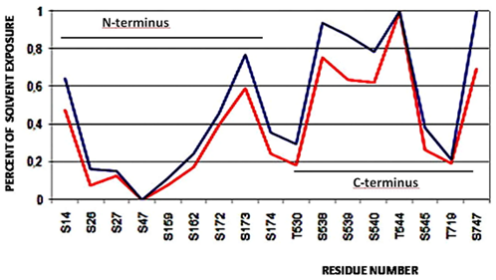
Analysis of phosphorilated sites. The Solvent accessibility profile for the residues belonging to putative phosphorilation site, valuated using Surface program (red line) and ASAview server (blue line). The number indicates the sequence position and the one letter code identifies the amino acid. The figure also reports the segmental position of residues in the protein.

Moreover the flexibility and the open structure of Sirt-1 termini could also favour the binding of phosphorylating proteins in order to activate regulation processes mediated by phoshorylation [3]. In fact, very recently the presence of phosphorylation sites located in the amino- and in carboxyl-terminal regions were found [3]. Therefore, in order to assess the ability of human Sirt-1 to be regulated by phorphorylation [43] and if there are the structural features inducing the interaction with phosphorylating proteins, we have evaluated the solvent accessibility of each residue, suggested as a putative site of phosphorylation [3]. Our data indicate that Ser14, Ser173, Ser538, Ser 539, Ser540, Thr 544 and Ser747 are largely exposed to the solvent (Figure 7) confirming that these residues have a high capability to be phosphorylated. If one considers the sequence position of Ser and Thr residues they are almost located in the N- and C- termini: Ser14, Ser26 and Ser27 are inserted in the segment 1–33 predicted by Anchor as a protein binding site. The residues Ser47 and Ser 159 are also included in the predicted segments 43–49 and 133–161, respectively, while the remaining Ser169, Ser173, Ser174, Thr196 and Thr 219 are very close to the above predicted segments. This suggests that these residues are actually involved in some way in the control of protein-protein binding and, as a consequence, in the control of the Sirt-1 function. The Sirt-1/AROS complex explains well this view by suggesting that the interaction between the two proteins occurs primarily through the involvement of the N-terminal segment with the achievement of greater compactness of the complex and the presence of changes that propagate to the active site in order to modulate the function. Work is in progress to simulate the presence of phosphate at various sites in the unordered regions in order to assess their structural or functional effects during the formation of the complex. The numerous and different structural features of unordered proteins such as conformational heterogeneity, secondary structural propensities, and tertiary contacts within disordered protein states can in principal generate many different interaction modes in proteins. Frequently these proteins mediate in signaling networks dynamic protein interactions that exhibit unusual binding characteristics, such as multisite dependence and ultrasensitivity. Such interactions are frequently modulated by phosphorylation, which requires disorder in the target protein both for optimal kinase accessibility and for subsequent accessibility of the binding motif [44], [45]. Therefore this binding appears to require multiple sites to be phosphorylated, suggesting a binding mode in which multiple phosphoepitopes engage a single receptor site in dynamic equilibrium. These observations allows to hypothesize a binding mode in which the multiple sites found in Sirt-1 might engage the putative receptor sites of AROS (5 Ser and 4 Thr residues) in a dynamic equilibrium. Moreover, Sirt-1 contains numerous additional phosphorylation sites remote from the targeting regions, making their participation in the complex unlikely. It is possible, however, that they may serve as decoy sites that compete with the key binding sites for phosphorylation by the targeting kinase [46].

Finally, a detailed study of the binding patches between AROS and Sirt-1 has shown that in Sirt-1 there is a conserved pharmacophore pocket composed by basic residues at the top of the cavity, acidic residues at its bottom and hydrophobic residues as edges. Moreover we have focused our attention on the presence of five aromatic residues (i.e. Trp176, Tyr185, Phe187, Trp221, Trp624) in the pocket that could be involved in putative stacking-interactions. These data could explain the high affinity of the allosteric site for synthetic activators containing aromatic rings and hydrophobic anchor points. Investigations are in progress to study the Sirt-1 interaction also with small allosteric effectors that have been recently identified [19].

## Methods

### Modelling of catalytic and allosteric sites of human Sirt-1

The three-dimensional model of catalytic site of human Sirt-1 (UniProt code: Q96EB6, region 244–498) was performed by comparative modelling strategy using the template structures of human Sirt-2 (PDB code: 1J8F chain A) [23], Hst2 from Saccharomyces cerevisae (PDB code: 1Q1A chain A) [21] and Sir2-Af1 from Archaeoglobus flugidus (PDB code: 1M2G chain A) [22] because the percentage of sequence identity between these proteins and human Sirt-1 was equal to 44%, 38% and 30%, respectively. Protein sequences were aligned with CLUSTALW [47]. The MODELLER9v5 program [48] was used to build 10 full-atom models of catalytic site, we used the ProsaII program to check the fitness of the sequences relative to the obtained structures and to assign a scoring function, and the PROCHECK program [49] to evaluate their stereochemical and structural packing quality. Secondary structures were assigned by the DSSP program [50]. Secondary structure predictions were performed with Jpred [29] server but structural class predictions were made by using PRECLASSPRO server [31]. The globularity of the best model was evaluated according to our recent work [27].

Moreover, the three-dimensional model of allosteric site of human Sirt-1 region 181–243 was performed using the template structure of a hexokinase from Sulfolobus Tokodaii by comparative modelling (PDB code: 2E2N) [51]. As the sequence identity between this Sirt-1 region and the homologous template model was lower than 30% (i.e. 26%), we used a procedure strategy in agreement with the rules recently reviewed to improve the quality of the modelling results at low target-template sequence similarity [52]. Allosteric and catalytic sites have been connected by using the Builder module of Insight II and the related model was subjected to molecular dynamics simulations.

### *Modelling of N-terminal and C-terminal regions of human Sirt-1*

The BLAST research [53] has found in databases only protein structures related to small sequences similar to the N-terminal and C-terminal regions of human Sirt-1 [region 1–180 and 499–747, respectively]. The prediction of unordered regions in the protein was made by Disopred server [36]. Moreover, a prediction of protein binding sites in the unordered regions was made by Anchor program that identifies specific binding regions undergoing disorder-to-order transition using a general disorder prediction method IUPred based on the assumption that disordered proteins have a specific amino acid composition that does not allow the formation of a stable well-defined structure [28]. The prediction of binding sites is based on estimating the energy content in free and in the bound states, and identifying segments that are potentially sensitive to these changes.

Therefore these regions were modelled by using a new approach combining fold recognition and comparative modelling. The preliminary models for N-terminal and C-terminal regions of Sirt-1 were obtained by fold recognition strategy using the Fugue and SAM-T06 servers [54]–[55]. Then, these models with the crystallographic structures suggested by BLAST were used as template for applying the comparative modelling strategy in order to obtain the complete models for both regions. In details, we used as template the following structures deposited in PDB: 1W36 (577–625), 1QHZ (1–34) for the N-terminal region and 1TYC (76–101) for C-terminal region.

In the obtained models the loop regions were refined using the LOOPY module of Jackal package [56]. LOOPY appeared to yield the best results for loop modelling, with models that are on average of 2–8% better than those generated by other programs [52]. The models obtained for N-terminal and C-terminal regions of Sirt-1 were subjected to molecular dynamics simulations.

### Molecular dynamics simulations

MD simulations were performed with GROMACS software package (v3.3.1) [57]. Models of different Sirt-1 regions were put in cubic boxes filled with SPC216 water molecules and GROMOS43a1 was selected as force-field. In order to optimize the system, the models were previously subjected to energy minimization and position restraints cycles. The simulations were carried out with periodic boundary conditions by adding sodium ions in order to the net electrostatic charge of the system is zero. The bond lengths were constrained by the all atoms LINCS algorithm. Particle Mesh Ewald (PME) algorithm was used for the electrostatic interactions with a cut-off of 0.9 nm, according to recent papers [29]. Simulations were conducted at neutral pH where the tritable groups of His, Glu and Asp residues are unprotonated. All simulations were run for 15 ns at room temperature (300 K) coupling the system to an external bath. GROMACS routines were utilized to check the trajectories and the quality of the simulations.

### Simulation of complete model of Sirt-1

The whole model of human Sirt-1 was obtained by comparing modelling using as template the models obtained for N-terminal region, allosteric site, catalytic site, and C-terminal region and the same procedure and programs described above. This model was minimized by using 500 steps of energy minimization under conjugate gradient algorithm to optimize side chain conformations and avoid sterical clashes according to the commonly used procedure [29], [58]–[59].

### Modelling and Simulation of Sirt-1/AROS complex

The three-dimensional model of AROS (UniProt code: Q86WX3, region 47–123) was performed by fold recognition strategy using the Fugue server [54]. The model obtained for AROS was subjected for 15 ns to molecular dynamics simulations using the protocol reported above to assess its conformational stability.

To simulate the Sirt-1/AROS complex we used the docking web server CLUSPRO [32] that resulted the best docking program in CAPRI experiments with a success rate of about 71% [32]. The models were selected by evaluating some features and parameters. The “Protein–Protein Interaction Server” [60] were used to identify the amino acids at the interface and to evaluate their solvent accessibility.

Moreover, the binding free energy between the different chains was calculated by using the DCOMPLEX program [33].

## Supporting Information

Table S1 Regions predicted as protein binding sites by Anchor program
(0.03 MB DOC)

Table S2 Analysis of interaction between AROS and Sirt-1 before and after MD
(0.03 MB DOC)

Table S3 Amino acid composition of Sirt-1
(0.18 MB DOC)

Figure S1 Details of catalytic groove before and after molecular dynamics are shown. We reported in pink and green the carbon atoms related to Sirt-1 before and after dynamics but N, O and H atoms always in blue, red and white, respectively. The Acetil-lysine, NAD and Sirt-1 residues are evidenced with labels.
(0.19 MB DOC)

Figure S2 Secondary structure of the whole Sirt-1 structure assigned by DSSP program. The N-terminal and C-terminal region sequences are reported in green and blue, respectively. The helices and beta-strands are indicated in red and cyan, respectively.
(0.07 MB DOC)

Figure S3 Flexibility plot for Sirt-1 sequence. Ordinate reports the value of Hydrophobicity x Volume obtained with a shifting window of 5 according to Ragone et al. Protein Eng. 1989 2(7):497–504. Abscissa reports the residue position.
(0.09 MB DOC)

Figure S4 Average area buried. Lower values indicates higher exposures of residues. The graph shows that the residues in the globular part of the protein are in average more buried than the N and C termini. In particular, residues in the N-terminus are in average more exposed.
(0.03 MB DOC)

Figure S5 The average molecular weight of residues with a shifting window of 5. The graph shows that the compact globular core is made in average of high molecular weight residues while the N- and C- termini are made of low molecular weight residues and thus smaller residues are located in the more fluctuating or flexible structural regions. It is interesting to note the highly fluctuating values in the C-terminal region in agreement with the presence of more structured segments in respect to the N-terminal region.
(0.03 MB DOC)

Figure S6 Ramachandran Plot
(0.46 MB DOC)
