# Supplementary material for: Human Sirt-1: Molecular Modeling and Structure-Function Relationships of an Unordered Protein
Source: PLoS One. 2009 Oct 8;4(10):e7350. doi: 10.1371/journal.pone.0007350 (PMC2753774; doi:10.1371/journal.pone.0007350)
Supplement: Table S1 — Regions predicted as protein binding sites by Anchor program (0.03 MB DOC) [file pone.0007350.s001.doc]

| **No.** | **Start** | **End** | **Length** |
| --- | --- | --- | --- |
| 1 | 1 | 33 | 33 |
| 2 | 43 | 49 | 7 |
| 3 | 53 | 125 | 73 |
| 4 | 133 | 161 | 29 |
| 5 | 184 | 193 | 10 |
| 6 | 220 | 225 | 6 |
| 7 | 498 | 503 | 6 |
| 8 | 521 | 528 | 8 |
| 9 | 549 | 574 | 26 |
| 10 | 588 | 593 | 6 |
| 11 | 618 | 624 | 7 |
| 12 | 637 | 672 | 36 |
| 13 | 691 | 706 | 16 |
| 14 | 710 | 744 | 35 |
