# Supplementary material for: Human Sirt-1: Molecular Modeling and Structure-Function Relationships of an Unordered Protein
Source: PLoS One. 2009 Oct 8;4(10):e7350. doi: 10.1371/journal.pone.0007350 (PMC2753774; doi:10.1371/journal.pone.0007350)
Supplement: Table S2 — Analysis of interaction between AROS and Sirt-1 before and after MD (0.03 MB DOC) [file pone.0007350.s002.doc]

|  | **Interface ASA** | **Residues of interaction** | **H-bonds** | **Salt-bridge** | **binding free energy** |
| --- | --- | --- | --- | --- | --- |
| Before MD | 973.9 | 30 | 1 | 29 | -10.19 |
| After MD | 1,257.49 | 38 | 8 | 46 | -21.41 |
