# Supplementary material for: Human Sirt-1: Molecular Modeling and Structure-Function Relationships of an Unordered Protein
Source: PLoS One. 2009 Oct 8;4(10):e7350. doi: 10.1371/journal.pone.0007350 (PMC2753774; doi:10.1371/journal.pone.0007350)
Supplement: Table S3 — Amino acid composition of Sirt-1 (0.18 MB DOC) [file pone.0007350.s003.doc]

N-Terminal Allosteric site +Catalytic Core C-Terminal


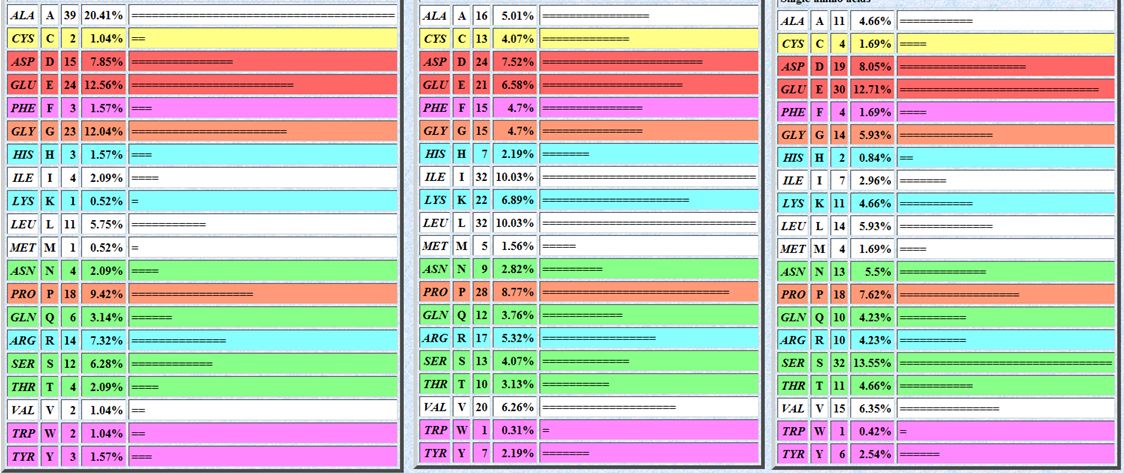


The table shows the amino acid composition of N-terminal, Allosteric site +Catalytic Core and C-terminal of Sirt-1. The central globular core of the protein has an amino acid composition reflecting that of an average globular protein. The two termini have in general a depletion of apolar amino acids (as Ile, Leu, Met, Val, Cys, Phe) and an increase of polar or small amino acids (as Glu, Gly, Ser, Ala). However, the comparison of the two terminal segments shows a quite different composition probably due to the presence of relatively more structured segments in the C-terminal region. A higher presence in these segments of amino acids with small volumes as Gly, Ser and Ala increases the local flexibilities and contributes to entropically stabilize the protein.

Color Code - Cyan: H, K, R (Polar positive); Red: D, E (Polar negative); Green: S, T, N, Q (Polar neutral); White: A, V, L, I, M (Non-polar aliphatic); Purple: F, W, Y (Non-polar aromatic); Brown: P, G; Yellow: C
