# Supplementary figures and images for: Human Sirt-1: Molecular Modeling and Structure-Function Relationships of an Unordered Protein
Source: PLoS One. 2009 Oct 8;4(10):e7350. doi: 10.1371/journal.pone.0007350 (PMC2753774; doi:10.1371/journal.pone.0007350)

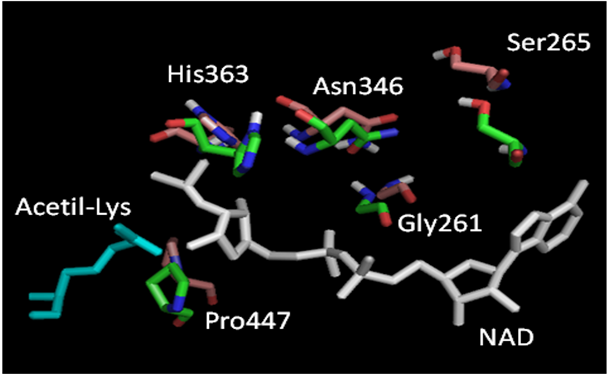

Supplement: Figure S1 — Details of catalytic groove before and after molecular dynamics are shown. We reported in pink and green the carbon atoms related to Sirt-1 before and after dynamics but N, O and H atoms always in blue, red and white, respectively. The Acetil-lysine, NAD and Sirt-1 residues are evidenced with labels. (0.19 MB DOC) [file pone.0007350.s004.doc]

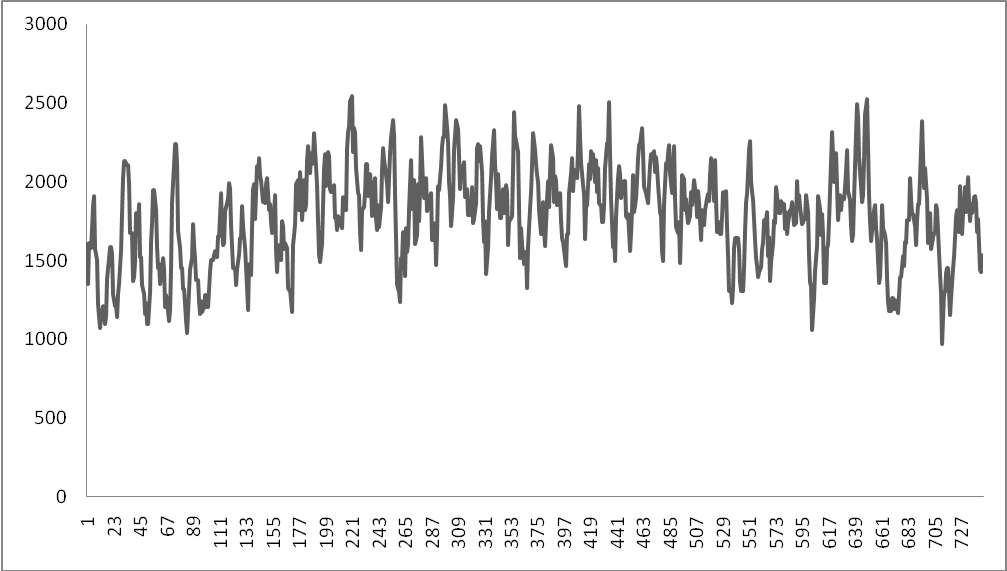

Supplement: Figure S3 — Flexibility plot for Sirt-1 sequence. Ordinate reports the value of Hydrophobicity x Volume obtained with a shifting window of 5 according to Ragone et al. Protein Eng. 1989 2(7):497–504. Abscissa reports the residue position. (0.09 MB DOC) [file pone.0007350.s006.doc]

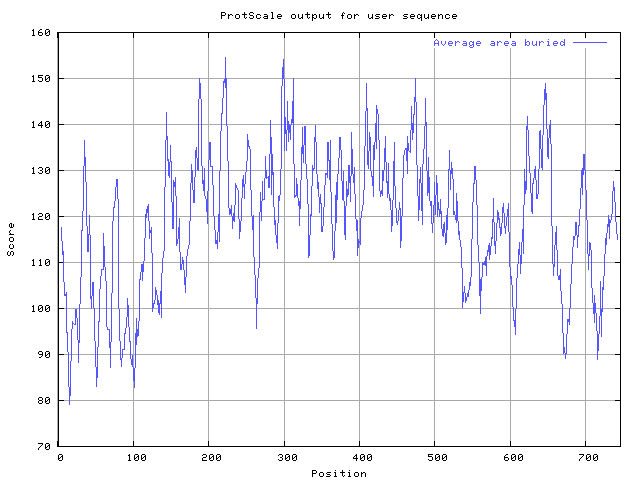

Supplement: Figure S4 — Average area buried. Lower values indicates higher exposures of residues. The graph shows that the residues in the globular part of the protein are in average more buried than the N and C termini. In particular, residues in the N-terminus are in average more exposed. (0.03 MB DOC) [file pone.0007350.s007.doc]

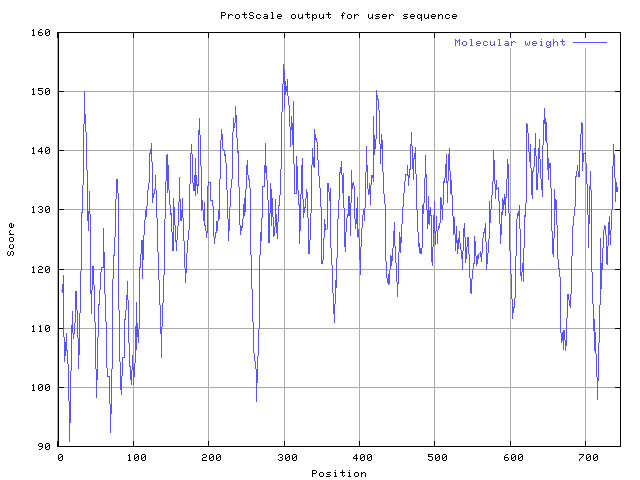

Supplement: Figure S5 — The average molecular weight of residues with a shifting window of 5. The graph shows that the compact globular core is made in average of high molecular weight residues while the N- and C- termini are made of low molecular weight residues and thus smaller residues are located in the more fluctuating or flexible structural regions. It is interesting to note the highly fluctuating values in the C-terminal region in agreement with the presence of more structured segments in respect to the N-terminal region. (0.03 MB DOC) [file pone.0007350.s008.doc]

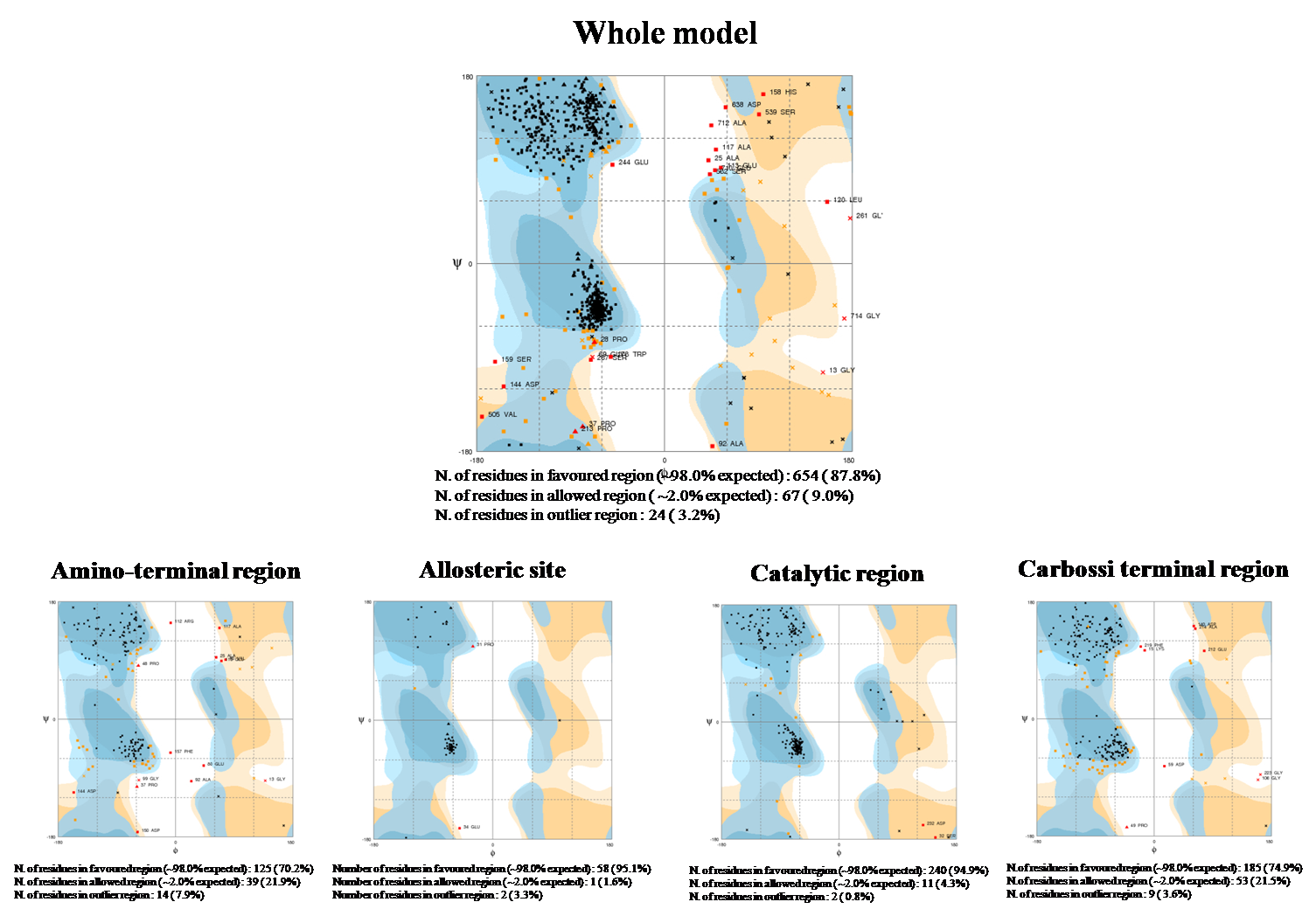

Supplement: Figure S6 — Ramachandran Plot (0.46 MB DOC) [file pone.0007350.s009.doc]
