# Supplementary material for: Human Sirt-1: Molecular Modeling and Structure-Function Relationships of an Unordered Protein
Source: PLoS One. 2009 Oct 8;4(10):e7350. doi: 10.1371/journal.pone.0007350 (PMC2753774; doi:10.1371/journal.pone.0007350)
Supplement: Figure S2 — Secondary structure of the whole Sirt-1 structure assigned by DSSP program. The N-terminal and C-terminal region sequences are reported in green and blue, respectively. The helices and beta-strands are indicated in red and cyan, respectively. (0.07 MB DOC) [file pone.0007350.s005.doc]

| Seq: | MADEAALALQ PGGSPSAAGA DREAASSPAG EPLRKRPRRD GPGLERSPGE | 50 |
| --- | --- | --- |
| SS: | **cHHHHHHTTS** **SSSSHHHHHH** **HHHTcccSSS** **cccEEccSSS** **STTcEEcScS** |  |
|  |  |  |
|  |  |  |
| Seq: | PGGAAPEREV PAAARGCPGA AAAALWREAE AEAAAAGGEQ EAQATAAAGE | 100 |
| SS: | **ScEEBcSSSS** **cBcSSccSSS** **ccHHHHHHSS** **SccccccccS** **ScScccBcSS** |  |
|  |  |  |
|  |  |  |
| Seq: | GDNGPGLQGP SREPPLADNL YDEDDDDEGE EEEEAAAAAI GYRDNLLFGD | 150 |
| SS: | **TTSSSccBSc** **EESccEEESS** **ccccSSSccc** **cHHHHHHHHS** **cEESSSEEcc** |  |
|  |  |  |
|  |  |  |
| Seq: | EIITNGFHSC ESDEEDRASH ASSSDWTPRP RIGPYTFVQQ HLMIGTDPRT | 200 |
| SS: | **HHHHHTTccS** **ccHHHHHHSS** **cccccScScc** **cTTHHHHHHH** **HHHHSSScHH** |  |
|  |  |  |
|  |  |  |
| Seq: | ILKDLLPETI PPPELDDMTL WQIVINILSE PPKRKKRKDI NTIEDAVKLL | 250 |
| SS: | **HHHHHHHHHc** **ccccTTTHHH** **HHHHHHHSSH** **HHHHHSGGGS** **cccTTHHHHH** |  |
|  |  |  |
|  |  |  |
| Seq: | QECKKIIVLT GAGVSVSCGI PDFRSRDGIY ARLAVDFPDL PDPQAMFDIE | 300 |
| SS: | **HHcSSEEEEE** **EcSSIIIIIS** **ccTTTSSSTT** **TTcSTTTTTc** **ccHHHHScHH** |  |
|  |  |  |
|  |  |  |
| Seq: | YFRKDPRPFF KFAKEIYPGQ FQPSLCHKFI ALSDKEGKLL RNYTQNIDTL | 350 |
| SS: | **HHHHccHHHH** **HHHHHScSSc** **cccccTTHHH** **HHHHHHTcEE** **EEEEccSScH** |  |
|  |  |  |
|  |  |  |
| Seq: | EQVAGIQRII QCHGSFATAS CLICKYKVDC EAVRGDIFNQ VVPRCPRCPA | 400 |
| SS: | **HHHSScSSEE** **ESSccSScEE** **ESSSccEEcH** **HHHHHHHHTT** **cccBcSSccT** |  |
|  |  |  |
|  |  |  |
| Seq: | DEPLAIMKPE IVFFGENLPE QFHRAMKYDK DEVDLLIVIG SSLKVRPVAL | 450 |
| SS: | **TcTTcBEEcS** **cccTTSSScH** **HHHHHcScSS** **cccSEEEEEE** **ScccSTTTTH** |  |
|  |  |  |
|  |  |  |
| Seq: | IPSSIPHEVP QILINREPLP HLHFDVELLG DCDVIINELC HRLGGEYAKL | 500 |
| SS: | **HHHHScTTcc** **EEEEESSccT** **TcSScEEEES** **cHHHHHHHHH** **HHSccccccc** |  |
|  |  |  |
|  |  |  |
| Seq: | CCNPVKLSEI TEKPPRTQKE LAYLSELPPT PLHVSEDSSS PERTSPPDSS | 550 |
| SS: | **cccSSccccc** **EEccSScccc** **HHHHHHSccc** **cccccccccc** **cccSSccccc** |  |
|  |  |  |
|  |  |  |
| Seq: | VIVTLLDQAA KSNDDLDVSE SKGCMEEKPQ EVQTSRNVES IAEQMENPDL | 600 |
| SS: | **ccEEccTTTT** **SSccIIIIII** **IIIEEcScSc** **SScccccSSB** **ccTTTTTTSc** |  |
|  |  |  |
|  |  |  |
| Seq: | KNVGSSTGEK NERTSVAGTV RKCWPNRVAK EQISRRLDGN QYLFLPPNRY | 650 |
| SS: | **cSSccccSSS** **cccccScHHH** **HHHHHHHScc** **ccSSBccccc** **EEEEcScSSS** |  |
|  |  |  |
|  |  |  |
| Seq: | IFHGAEVYSD SEDDVLSSSS CGSNSDSGTC QSPSLEEPME DESEIEEFYN | 700 |
| SS: | **ccBTTTBccc** **cSScHHHHHT** **TcccSSSEEE** **EcccccHHHH** **HSHHHHHHHc** |  |
|  |  |  |
|  |  |  |
| Seq: | GLEDEPDVPE RAGGAGFGTD GDDQEAINEA ISVKQEVTDM | 740 |
| SS: | **cccSScSSSS** **cSBcSSSScS** **SScccBSccH** **HHHHHHHHHc** |  |
|  |  |  |
|  |  |  |
